# Dual-pool, three-phase kinetic model of anaerobic digestion in batch mode^☆^

**DOI:** 10.1016/j.heliyon.2022.e09194

**Published:** 2022-03-26

**Authors:** Bruno Gouveia, Elizabeth Duarte, Aires dos Santos, Edgar Fernandes

**Affiliations:** a IN+ Centre for Innovation, Technology and Policy Research, LARSyS, Instituto Superior Técnico, Universidade de Lisboa, Av. Rovisco Pais, 1, 1049-001 Lisbon, Portugal; b LEAF - Linking Landscape, Environment, Agriculture and Food, Instituto Superior de Agronomia, Universidade de Lisboa, Tapada da Ajuda, 1349-017 Lisbon, Portugal; c MARETEC/DEM – Marine, Environment and Technology Centre, LARSyS, Instituto Superior Técnico, Universidade de Lisboa, Av. Rovisco Pais, 1, 1049-001 Lisbon, Portugal

**Keywords:** Kinetic model, Anaerobic digestion, Bioreactor, Biogas production, Batch mode

## Abstract

The ability of anaerobic digestion to create value from waste gives it an important role in reducing greenhouse gas emissions and in the transition to a circular economy. For a better understanding of the digestion process and in order to reduce the number of time-consuming batch tests, an analytical model was developed to describe the kinetics of biogas production. Assuming that the organic fraction of the substrate has different degradation rates, the whole process was modelled as two groups of 1^st^ order reactions.

The model was tested with published data and showed an excellent performance in reproducing the experimental information. Moreover, its kinetic constants provided a useful insight into the internal processes of anaerobic digestion and the substrate characteristics. Given its accuracy in fitting the data, the model can be used as an auxiliary tool to determine the biogas potential, presenting itself as the most complete empirical model currently available.

## Highlights

• Original model based on the kinetics of the digestion process.
• Better performance than current empirical approaches.
• Interesting tool to reduce the duration of batch tests.

## 1 Introduction

The reduction of greenhouse gas (GHG) emissions and the fight against climate change constitute one of the greatest challenges of the modern world. In this context, anaerobic digestion (AD) presents itself as a promising technology, contributing to the achievement of the goals defined in the Paris Agreement (Paolini et al., 2018). By transforming organic matter into energy and natural fertilizers, waste that would once have been deposited into landfills (releasing GHGs) is continuously reused. Anaerobic digestion is thus a waste valorization process, whose principles are directly connected to the basic concepts of a circular economy (Martin and Parsapour, 2012).

**Figure 1:**
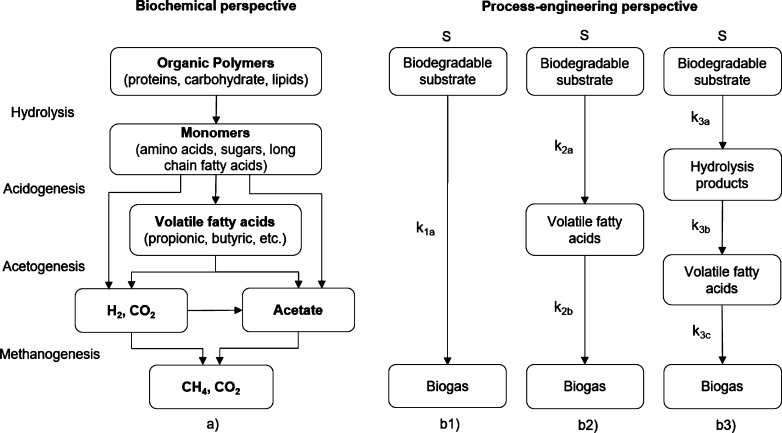
Reaction steps that occur during anaerobic degradation of the organic fraction of the substrate from a biochemical perspective: a) simplified scheme adapted from Shin and Song (1995); and from a process-engineering perspective: b1) 1^st^ order, single-phase model; b2) 1^st^ order, two-phase model; b3) 1^st^ order, three-phase model.

Anaerobic digestion consists in a heterogeneous ecosystem where several groups of microorganisms participate interactively in the conversion of complex organic matter into biogas. From a biochemical point of view, the AD can be described in 4 phases: hydrolysis, acidogenesis, acetogenesis and methanogenesis (Appels et al., 2008; Shin and Song, 1995; Lyberatos and Skiadas, 1999). The process is schematically represented in Fig. 1. The biogas produced during the process consists essentially of a mixture of 50-70% methane (CH_4_) and 30-50% carbon dioxide (CO_2_), depending on the substrate and the AD process selected (Rasi et al., 2007; Fagerström et al., 2018).

With the growing interest and investment in the biogas production industry, modelling is paramount for a better understanding of the anaerobic digestion process (Gerber and Span, 2008; Gustafsson et al., 2020). The determination of the degradation kinetics and the maximum volume of biogas that can be extracted from a given substrate is essential in reactor design and performance control (Adl et al., 2015). These parameters are obtained from anaerobic digestion batch assays, which can be quite time-consuming due to long retention times. Therefore, the construction of fitly calibrated mathematical models can provide an alternative in this sense, reducing the number of tests and allowing a better insight into the system's behaviour (Dursun et al., 2011).

Taking this into consideration and in order to develop an analytical model that would describe the kinetics of substrate degradation during anaerobic digestion, a review of the available models was initially performed. In the following section the different classes of models are analysed.

### 1.1 Kinetic models of batch AD

The existing analytical models of AD can be divided into two groups: mechanistic or empirical. Mechanistic models such as ADM1 developed by the International Water Association (Batstone et al., 2002), allow the simulation of bacterial growth and the biochemical reactions of the process. However, these models are complex and quite difficult to calibrate as they require a high number of input parameters and experimental measurements that, in most cases, are not performed in AD facilities (Gerber and Span, 2008). In this regard, empirical models (less sophisticated) have been the object of study by several researchers (Gerber and Span, 2008; Vavilin et al., 2008; Lauwers et al., 2013; Kythreotou et al., 2014). With fewer experimental measurements, empirical models such as: the 1^st^ order kinetic models (Veeken and Hamelers, 1999; Turick et al., 1991) or the Modified Gompertz model (Zwietering et al., 1990), allow to describe the kinetics of the digestion process in batch mode and thus estimate the biogas potential for any type of substrate.

In processes where the hydrolysis is the rate-limiting step, the saturation effects of the bacterial growth rate due to the limited presence of nutrients in the substrate can be neglected, and the substrate degradation follows a 1^st^ order kinetics (Brulé et al., 2014), according to equation (1):

(1) $$\frac{dS_{t}}{dt}=-k\cdot S_{t}$$

Where *k* is the degradation rate of the organic matter and $S_{t}$ the concentration of the organic fraction of the substrate at the instant *t*. Thus, from a process-engineering perspective, the complex system of biochemical and biological interactions that constitutes the AD can be simplified in a mechanism of one or more phases, described by 1^st^ order kinetic reactions, as schematically represented in Fig. 1.

The 1^st^ order, single-phase model is the most frequently used and describes the degradation of the organic fraction of the substrate in a single reaction. The cumulative biogas production function associated to this model can be expressed by equation (2):

(2) $$S_{t}=S\left(1-e^{-k_{1a}t}\right)$$

Where *S* is the initial organic matter concentration of the substrate and $k_{1a}$ the kinetic constant associated with the conversion of the organic fraction of the substrate into biogas. This model has been applied by several authors (El-Mashad, 2013; Angelidaki et al., 2009; Jokela et al., 2005; Rao and Singh, 2004) and allows to obtain a reasonable estimate of the temporal progression of biogas produced.

Generally, multiphase models permit to describe the temporal conversion of the substrate into biogas with more detail, providing additional information on the behaviour of intermediate products formed during AD. Shin and Song (1995) proposed a model where the AD process is described in two consecutive phases of 1^st^ order reactions: acidification and methanation. During the acidification of the biodegradable fraction of the substrate, volatile fatty acids (VFA) are produced, which are then converted into biogas in the final stage of methanation (Demirel and Yenigün, 2002), according to equation (3):

(3) $$S_{t}=S\left[1+\frac{k_{2a}e^{-k_{2b}t}-k_{2b}e^{-k_{2a}t}}{k_{2b}-k_{2a}}\right]$$

Where $k_{2a}$ and $k_{2b}$ are the kinetic constants associated with the acidification and methanation steps, respectively.

In the scope of waste treatment from the bakery industry, Deveci and Çiftçi (2001) developed a model where the AD is considered a 3-phase system. For this model, the cumulative production of biogas over time is expressed by equation (4):

(4) $$S_{t}=S[1-e^{-k_{3a}t}-k_{3a}\frac{e^{-k_{3a}t}-e^{-k_{3b}t}}{k_{3b}-k_{3a}}-k_{3a}k_{3b}\frac{\left(k_{3c}-k_{3b}\right)e^{-k_{3a}t}-\left(k_{3c}-k_{3a}\right)e^{-k_{3b}t}+\left(k_{3b}-k_{3a}\right)e^{-k_{3c}t}}{\left(k_{3b}-k_{3a}\right)\left(k_{3c}-k_{3a}\right)\left(k_{3c}-k_{3b}\right)}]$$

Where $k_{3a}$, $k_{3b}$ and $k_{3c}$ are the kinetic constants associated with the conversion of biodegradable substrate into hydroylis products, the hydrolysis products into VFA and VFA into biogas, respectively. This three-phase system was also applied by Safari et al. (2011), in the scope of leachate treatment from municipal waste.

**Figure 2:**
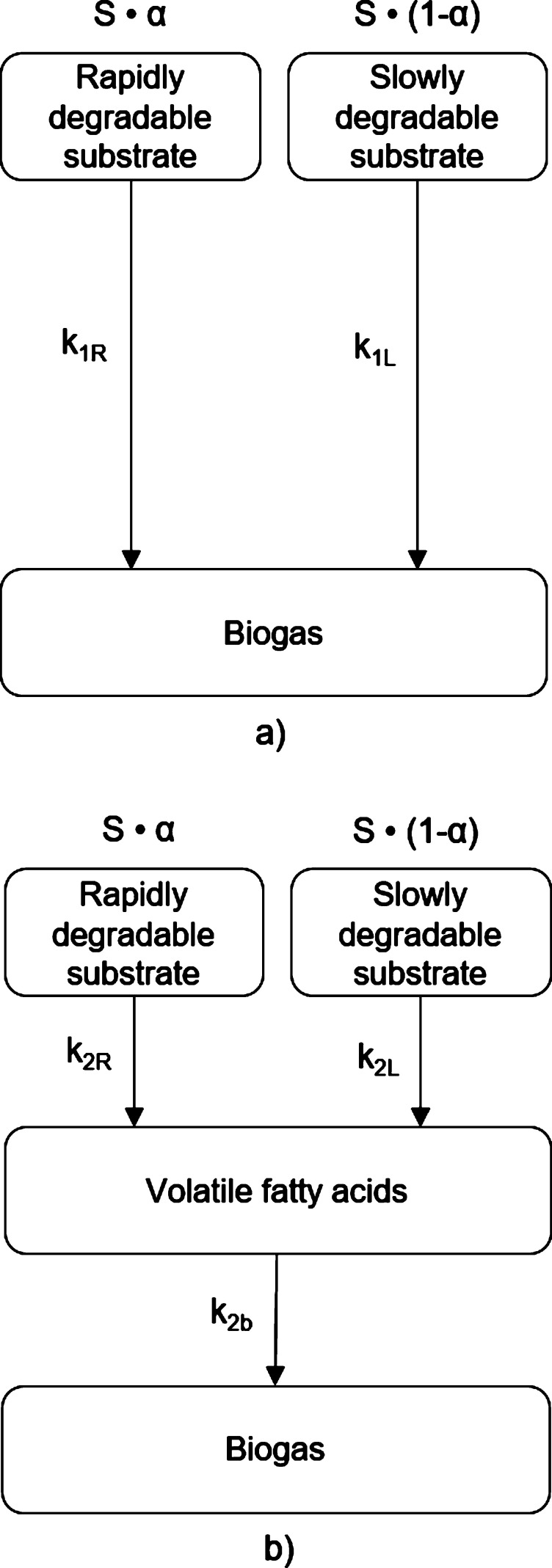
Reaction steps assuming that the substrate has different degradation rates: a) 1^st^ order, single-phase model; b) 1^st^ order, two-phase model.

The models previously mentioned assume that all organic matter degrades at the same rate. However, the chemical composition of substrates is typically heterogeneous and therefore the organic matter has different conversion velocities (Dijkstra et al., 2005). Simpler monomeric compounds are readily available to be biodegraded by heterotrophic microorganisms, whereas complex organic polymers require an extracellular breakdown (hydrolysis) before being biodegraded (Mertens et al., 2005). Having that in mind, Rao et al. (2000) and Kusch et al. (2008) described the AD process through a 1^st^ order model where the substrate was divided into two groups of components: rapidly biodegradable and slowly biodegradable. This approach is schematically represented in Fig. 2 and the respective cumulative function of biogas production is given by equation (5):

(5) $$S_{t}=S\left[1-\alpha e^{-k_{1R}t}-(1-\alpha )e^{-k_{1L}t}\right]$$

Where *α* corresponds to the fraction of rapidly biodegradable substrate, and $k_{1R}$ and $k_{1L}$ are the kinetic constants associated with the conversion of rapidly and slowly degradable components of the substrate into biogas, respectively. Brulé et al. (2014) combined this approach with the 2-phase model proposed by Shin and Song (1995), resulting in equation (6):

(6) $$S_{t}=S[\alpha (1+\frac{k_{2R}e^{-k_{2b}t}-k_{2b}e^{-k_{2R}t}}{k_{2b}-k_{2R}})\;+(1-\alpha )(1+\frac{k_{2L}e^{-k_{2b}t}-k_{2b}e^{-k_{2L}t}}{k_{2b}-k_{2L}})]$$

Where $k_{2R}$ and $k_{2L}$ are the kinetic constants associated with the conversion of rapidly and slowly degradable components of the substrate into VFA.

The introduction of the concept that the organic fraction of the substrate has different degradation speeds, seems to improve the prediction of substrate degradation kinetics over time (Rao et al., 2000; Brulé et al., 2014).

As mentioned above, another empirical model widely used in the literature to analyse data related to bacterial population growth is the Modified Gompertz model (Donoso-Bravo et al., 2010; Li et al., 2018; Kafle and Kim, 2013; Browne et al., 2014; Tsapekos et al., 2018; Deepanraj et al., 2015). The Modified Gompertz model proposed by Zwietering et al. (1990) is a re-parameterisation of the traditional cumulative Gompertz model (Tjørve and Tjørve, 2017) and it can be express by equation (7):

(7) $$S_{t}=P\exp\left[-\exp\left(\frac{\mu \cdot e}{P}(\lambda -t)+1\right)\right]$$

Where *P* is the maximum biogas production, *μ* the maximum biogas production rate and *λ* the lag phase for biogas production to begin.

Typically, the determination of the parameters of all the models presented before, requires only experimental information regarding biogas production profiles. However, the values obtained for the kinetic constants are rarely analysed from a physical perspective of the process, thus lacking validation (Weinrich et al., 2018).

## 2 Model development

**Figure 3:**
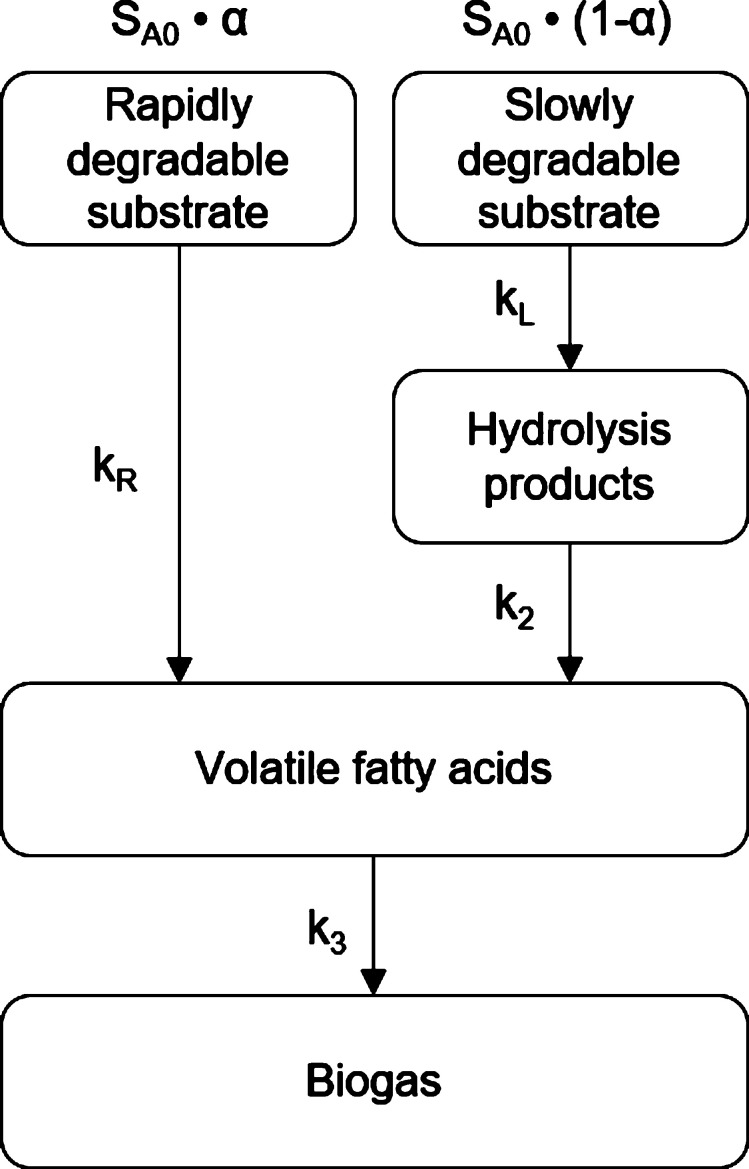
Schematic representation of the proposed kinetic model of anaerobic digestion.

After a revision of the empirical models proposed by different researchers to characterize the AD process, a more complete kinetic model was developed with the objective of describing the temporal degradation of the substrate along the different phases in a more precise way and in order to obtain more realistic estimates for the maximum potential of biogas production. The proposed model is schematically represented in Fig. 3, where the following assumptions were adopted:

1. Organic matter has different conversion speeds and therefore the substrate can be divided into two major groups of components: rapidly and slowly biodegradable.
  Considering the approach of Brulé et al. (2014) and Rao et al. (2000), the total amount of rapidly biodegradable components ($C_{R}$) and slowly biodegradable components ($C_{L}$) can be expressed mathematically by equation (8) and (9), respectively:
  (8) $$C_{R}=\alpha \cdot S_{A0}$$
  (9) $$C_{L}=(1-\alpha )\cdot S_{A0}$$
  Where $S_{A0}$ is the initial concentration of biodegradable substrate and *α* the fraction of rapidly biodegradable substrate;
2. The process associated with the degradation of the slowly biodegradable fraction is simplified in a system of 3 phases: hydrolysis, acidogenesis and methanogenesis, according to Deveci and Çiftçi (2001). Whereas the process associated with the rapidly biodegradable fraction is simplified into a system of only 2 phases, since this fraction is associated with the simplest monomeric compounds. $S_{A}$, $S_{B}$, $S_{C}$ and $S_{D}$ correspond to the concentrations of biodegradable substrate, products of hydrolysis, volatile fatty acids and biogas, respectively;
3. Considering that both H_2_ and CO_2_ are rapidly consumed by methanogenic bacteria, their role as intermediate products was neglected (Karakashev et al., 2006);
4. The reactions are irreversible and follow first order kinetics, which implies that the saturation effects are neglected. $k_{R}$, $k_{L}$, $k_{2}$ and $k_{3}$ are first order kinetic constants and correspond to the conversion of rapidly degradable components of the substrate into VFA, the conversion of slowly degradable components of the substrate into hydrolysis products, the conversion of the hydrolysis products associated with the slowly degradable fraction into VFA, and the conversion of total VFA into biogas, respectively;
5. Due to the complexity and variability of substrate composition, mass balances are based on the chemical oxygen demand unit (COD) (Deveci and Çiftçi, 2001; Shin and Song, 1995).

With these assumptions, the kinetics of substrate degradation over the different stages of the process is expressed by the following set of differential equations ((10a), (10b) and (10c)):

(10a) $$\frac{dS_{A}}{dt}=-\left(\alpha k_{R}S_{A}+(1-\alpha )k_{L}S_{A}\right)$$

(10b) $$\frac{dS_{B}}{dt}=(1-\alpha )\left(k_{L}S_{A}-k_{2}S_{B}\right)$$

(10c) $$\frac{dS_{C}}{dt}=k_{2}S_{B}(1-\alpha )+\alpha k_{R}S_{A}-k_{3}S_{C}$$

In order to obtain $S_{A}$, $S_{B}$, $S_{C}$ and $S_{D}$ concentrations as a function of time, the differential equations previously presented were integrated. The degradation of the organic fraction of the substrate over time is thus expressed by equation (11):

(11) $$S_{A}=S_{A0}\left(\alpha e^{-k_{R}t}+(1-\alpha )e^{-k_{L}t}\right)$$

The intermediate function associated with the rate of accumulation of hydrolysis products from the rapidly degradable fraction of the substrate is described by equation (12):

(12) $$S_{B}=S_{A0}\left[(1-\alpha )k_{L}\frac{e^{-k_{L}t}-e^{-k_{2}t}}{k_{2}-k_{L}}\right]$$

Assuming that the initial concentration of volatile fatty acids is zero, $S_{C}\left(t=0\right)=0$, the accumulation of VFA as a function of time is described by equation (13):

(13) $$S_{C}=S_{A0}[\alpha k_{R}\frac{e^{-k_{R}t}-e^{-k_{3}t}}{k_{3}-k_{R}}\;\;+(1-\alpha )k_{L}k_{2}\frac{\left(k_{3}-k_{2}\right)e^{-k_{L}t}-\left(k_{3}-k_{L}\right)e^{-k_{2}t}+\left(k_{2}-k_{L}\right)e^{-k_{3}t}}{\left(k_{2}-k_{L}\right)\left(k_{3}-k_{L}\right)\left(k_{3}-k_{2}\right)}]$$

The $S_{C}$ function is of particular interest because it allows to describe the accumulation of volatile fatty acids along the process. The concentration of VFA should be closely monitored because these compounds can inhibit the development of methanogenic bacteria, due to changes in the pH of the medium (He et al., 2006). Finally, the biogas concentration results from the mass balance of the components involved in anaerobic digestion (equation (14)):

(14) $$S_{D}=S_{A0}-S_{A}-S_{B}-S_{C}$$

Assuming that the whole substrate is converted into biogas, the maximum potential for biogas production is equal to the initial substrate concentration for an infinite retention time: $S_{A0}=S_{max}$. Thus, the cumulative biogas production function is expressed by equation (15):

(15) $$S_{D}=S_{max}[\alpha (1-e^{-k_{R}t}-k_{R}\frac{e^{-k_{R}t}-e^{-k_{3}t}}{k_{3}-k_{R}})\;+(1-\alpha )(1-e^{-k_{L}t}-k_{L}\frac{e^{-k_{L}t}-e^{-k_{2}t}}{k_{2}-k_{L}}\;\;-k_{L}k_{2}\frac{(k_{3}-k_{2})e^{-k_{L}t}-(k_{3}-k_{L})e^{-k_{2}t}+(k_{2}-k_{L})e^{-k_{3}t}}{\left(k_{2}-k_{L})(k_{3}-k_{L})(k_{3}-k_{2}\right)})]$$

The final $S_{D}$ function, which describes the cumulative production of biogas over time and its maximum potential, is thus composed of 6 parameters.

### 2.1 Parameter determination

In order to ensure realistic simulations, the 6 parameters of the cumulative biogas production function ($S_{D}$) should be adjusted according to the substrate used in the batch anaerobic digestion test. The unknown parameters can be determined using a numerical optimization procedure (Isermann and Münchhof, 2010). However, it is important to point out that the choice of the algorithm, objective function and initial conditions influences the value of the estimated parameters and the accuracy of the model.

In order to initialise the optimization procedure it is necessary to provide an initial estimate for the model parameters. Considering that the problem in question is non-convex, a methodology was established according to the following steps, where the initial estimate for the parameters of the proposed model was provided by the simplest 1^st^ order model:

1. Set random initial conditions for the parameters of the 1^st^ order, single-phase model (S=x and $k_{1a}=y$).
2. Run the optimization algorithm until the final estimate for the parameters of the simplest model is obtained ($S^{x}$ and $k^{y}$). The parameters obtained for this model will always be the same, regardless of the initial conditions chosen.
3. Use the values obtained in step 2 as the basis for the initial estimate of the parameters of the proposed model, according to Table 1.

**Table 1:** Selected initial conditions for the model parameters.

Model	Parameters					
1^st^ order, single-phase	*S*	*k*_1*a*_				
	x	y				
Proposed model	*S*_*max*_	*k*_*L*_	*k*_*R*_	*k*_2_	*k*_3_	*α*
	*S*^*x*^	*k*^*y*^/2	*k*^*y*^	*k*^*y*^ × 2	*k*^*y*^ × 3	0.5

In this study, the unknown parameters were determined using the Levenberg-Marquardt algorithm (Lourakis et al., 2005; Moré, 1978), which was executed in Matlab® software version 9.5.0 (R2018b) through the non-linear optimization function [lsqcurvefit]. This algorithm was chosen due to its suitability to solve non-linear optimization problems. In this case, a least squares problem, where the set of unknown parameters (*η*) were iteratively determined by minimizing the objective function *ψ* (given by equation (16)), which measures the square of the difference between the experimental values ($y_{exp}$) and the values predicted by the model ($S_{D}$):

(16) $$\psi (\eta )=\min\sum_{t=0}^{N}{\left[y_{exp}(t)-S_{D}(t,\eta )\right]}^{2}$$

Where *N* is the number of experimental measurements performed. The optimization process ended when the variation in the residual was less than the specified tolerance of $1\times 10^{-6}$.

## 3 Results & discussion

In order to evaluate the ability of the developed model to reproduce the experimental data in a realistic and accurate way, three case studies with published experimental data were analysed.

### 3.1 Case study 1

The first case study aimed to evaluate not only the ability of the model to describe the biogas production kinetics, but also the realism of the estimate reproduced for the VFA accumulation profile, obtained from the model's mass balances.

Experimental data regarding biogas production from municipal solid waste (MSW) were collected from the work published by Rao et al. (2000). The batch tests were conducted under room temperature conditions (26 ± 4 °C), in a reactor with a working volume of 2 L.

**Figure 4:**
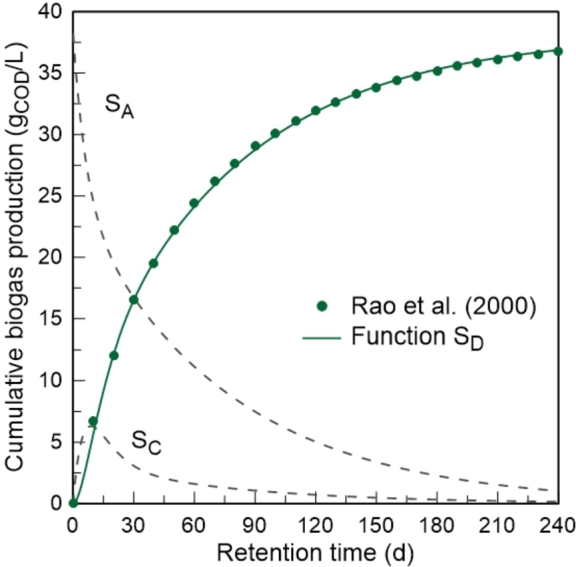
Graphical representation of the model performance, resulting from the simulation between the *S*_*D*_ function and experimental data of biogas production published by Rao et al. (2000).

The optimal solution found for the model parameters, resulting from the simulation between the $S_{D}$ function and the experimental data, was the following: $S_{A0}=38.1625$ g_COD_/L; $k_{L}=0.0133$ d^-1^; $k_{R}=0.1532$ d^-1^; $k_{2}=0.1274$ d^-1^; $k_{3}=0.1181$ d^-1^; and α=0.3532. As expected, given the heterogeneity of the substrate in question (municipal waste), the kinetic constant $k_{L}$ indicates the lowest value and therefore hydrolysis is the rate-limiting step of the process. The graphical representation of this simulation is shown in Fig. 4, where it is possible to observe that the $S_{D}$ function accurately reproduces the kinetics of biogas production. The coefficient of determination ($R^{2}$) is approximately 0.999. Therefore, it can be concluded that the choice of using 1^st^ order reactions to describe the different phases of the digestion process was appropriate.

**Figure 5:**
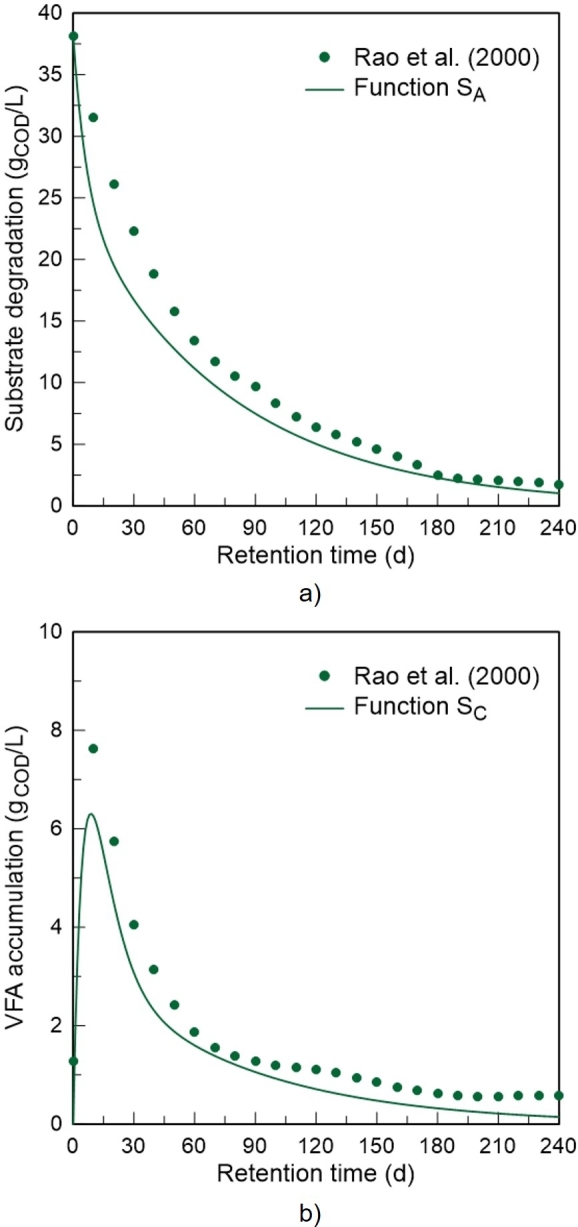
Graphical comparison between model predictions and the respective experimental data published by Rao et al. (2000): a) substrate degradation profile; b) volatile fatty acid accumulation profile.

The dashed lines correspond to the substrate degradation ($S_{A}$) and volatile fatty acid accumulation ($S_{C}$) profiles, obtained from the same set of parameters. These profiles present a typical pattern for batch anaerobic digestion (Shin and Song, 1995; Mottet et al., 2009). However, from Fig. 4 alone it is not possible to guarantee that these profiles describe the experimental information with the same accuracy. Taking this into account, the estimates produced by the $S_{A}$ and $S_{C}$ functions of the developed model were compared with the respective experimental data. For this purpose, VFA were considered as acetic acid and the data associated with the concentration of acetic acid were converted using the theoretical conversion coefficient: $Y_{conv}=1.07g_{O_{2}}/g_{CH_{3}COOH}$, obtained from the oxidation reaction of acetic acid. The simulation results are graphically represented in Fig. 5.

As can be observed, the proposed substrate degradation and volatile fatty acid accumulation functions show concordance with the experimental results, confirming the potential of the model. The $S_{A}$ function presents a coefficient of determination of 0.973, while for the $S_{C}$ function, $R^{2}=0.980$.

The model seems to produce estimates consistent with the experimental results. However, for a better understanding of the behaviour of its parameters, two more case studies were analysed.

### 3.2 Case study 2

**Figure 6:**
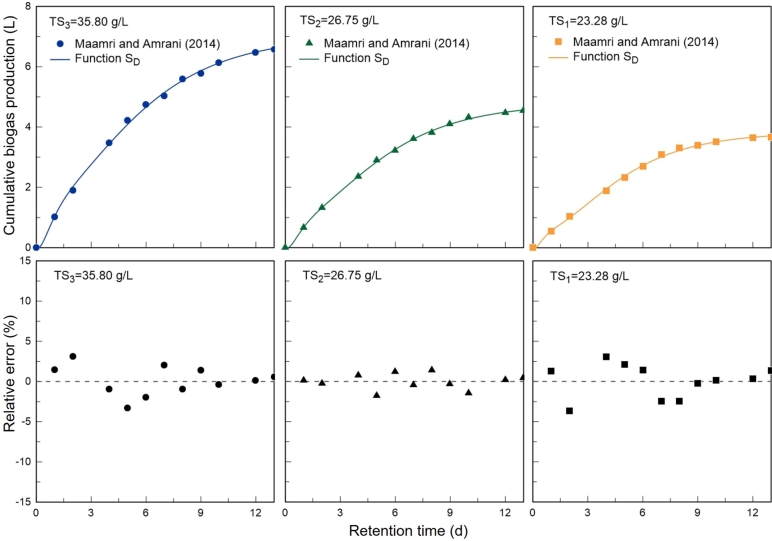
Graphical representation of the model performance, resulting from the simulation between the *S*_*D*_ function and experimental data of biogas production published by Maamri and Amrani (2014) (top). Relative error expressed by (*S*_*D*_ − *y*_*exp*_)/*y*_*exp*_ (bottom).

In order to assess the consistency of the parameters estimated by the optimisation algorithm, in the second case study it was analysed the kinetics of biogas production of waste activated sludge, generated in wastewater treatment plants (WWTPs). Experimental data were collected from the work published by Maamri and Amrani (2014). The batch tests were conducted under thermophilic conditions (55 °C), in a reactor with a working volume of 4.5 L. The simulation results between the developed model and the experimental data are presented in Fig. 6, where 3 different amounts of total solids (TS) were analysed in order to understand their effect on the kinetic constants of the model.

It can be observed that the $S_{D}$ function accurately reproduces the kinetics of biogas production. The coefficient of determination is approximately 0.999 for the 3 tests, which means that the model can explain about 99.9% of the variability of the experimental information.

The relative error between the model and the experimental data, defined as the quotient between the residual ($S_{D}-y_{exp}$) and $y_{exp}$, never exceeds 3.64% (maximum value found for TS_1_=23.28 g/L) and oscillates around 0 over the 3 tests, as can be seen in Fig. 6. Taking into account that the empirical model developed is an approximation of a complex system of biochemical processes, the results obtained are quite satisfactory.

#### 3.2.1 Parameter interpretation

The parameters of the model, obtained with the considered nonlinear optimization algorithm, are shown in Table 2. From these values, the following conclusions can be drawn:

1. The $S_{max}$ value identified by the model is higher than the last value measured experimentally for the 3 batch tests. The estimate for the maximum potential of biogas produced is therefore a reliable approximation. According to Weinrich et al. (2018), the biogas potential is the basis for the analysis of the performance of biogas plants.
2. Biogas production started almost immediately, which indicates the presence of rapidly biodegradable components. The hierarchy of values registered for parameter *α* is coherent. Taking into account that the same type of substrate was used in all tests, it would be expected that for compositions with higher TS there would be a higher concentration of rapidly degradable components.
3. The parameter $k_{L}$ registers the lowest value among the kinetic constants in every test, and therefore hydrolysis is the rate-limiting step of the process. In the presence of solid substrates like the one we are dealing with (waste activated sludge), hydrolysis often corresponds to the slowest stage of the process (Vavilin et al., 2008).
4. The kinetic constants ($k_{L}$, $k_{R}$, $k_{2}$ and $k_{3}$) decrease with the increase of the total solids concentration. The decrease in the overall kinetics of biogas production verified throughout the 3 tests is explained by the fact that the amount of biodegradable organic matter is successively higher. With more organic matter available, more time is needed to achieve the same percentage of degradation, which leads to a slower process (Koch and Drewes, 2014). According to Brulé et al. (2014), in 1^st^ order models the parameters do not necessarily describe the growth rate of the bacteria but rather the kinetics of substrate degradation.

**Table 2:** Optimal solution obtained for the parameters of the developed model and coefficient of determination for each test. Simulation of the *S*_*D*_ function with the experimental data from Maamri and Amrani (2014).

	Experimental tests		
	TS_1_	TS_2_	TS_3_
*S*_*max*_ (*L*)	3.818	4.775	6.998
*k*_*L*_ (*d*^−1^)	0.421	0.340	0.301
*k*_*R*_ (*d*^−1^)	3.262	2.139	1.932
*k*_2_ (*d*^−1^)	0.422	0.417	0.413
*k*_3_ (*d*^−1^)	3.262	2.314	2.180
*α*	0.221	0.265	0.289
*R*^2^	0.999	0.999	0.999

Although the identified parameters reproduce an estimate consistent with the experimental data, they should be reviewed for a larger number of tests. Given the variability of the chemical composition of the mixtures inside the reactor, it is necessary to define a reasonable range of values for the constants in order to take these variations into account.

### 3.3 Case study 3

**Figure 7:**
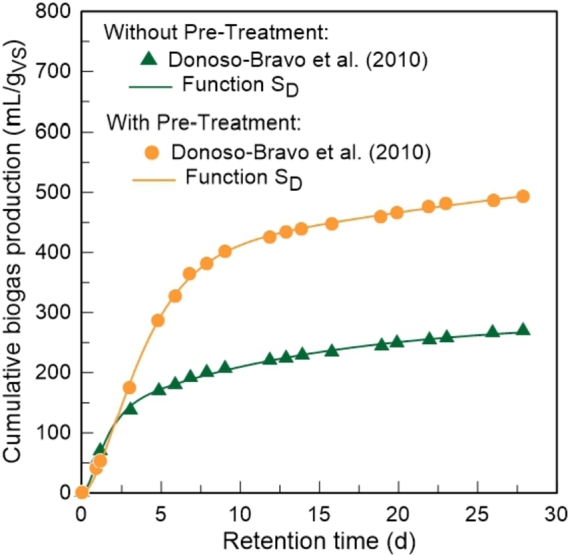
Simulation results between the *S*_*D*_ function of the developed model and experimental data published by Donoso-Bravo et al. (2010).

In the third case study the developed model was compared with some of the empirical models mentioned in the introductory section. For this purpose, it was analysed the effect of thermal pre-treatment (175 °C, 30 min) on the kinetics of biogas production of secondary sludge from municipal WWTPs. Experimental data were collected from the work published by Donoso-Bravo et al. (2010). The batch tests were carried out in glass bottles with a liquid volume of 120 mL, under mesophilic conditions (35 °C). The simulation results between the $S_{D}$ function and the experimental data, with and without pre-treatment, are represented in Fig. 7.

In both cases, the model describes the experimental information with high precision, being able to follow the kinetics of biogas production throughout the process. Once again, the coefficient of determination is around 0.999 for both tests.

#### 3.3.1 Parameter interpretation

The parameters of the model, obtained with the considered optimization algorithm, are presented in Table 3. From these values, the following conclusions can be drawn:

1. With the introduction of pre-treatment there was a significant increase in biogas production. The biogas potential for the test with pre-treatment almost doubled compared to the test where no pre-treatment was performed. Once again, the $S_{max}$ value estimated by the model is higher than the last value measured experimentally for the 2 cases.
2. The *α* parameter increased about 40% with the pre-treatment. This is explained by the fact that thermal pre-treatment causes the solubilization of the particulate material, and therefore a greater amount of organic matter is available to be immediately converted into biogas.
3. The parameter $k_{L}$ registers the lowest value among the kinetic constants in every test, and therefore hydrolysis is the rate-limiting step of the process. The presence of solid particles in the sludge makes hydrolysis the slowest stage of the anaerobic digestion process.
4. The kinetic constants ($k_{L}$, $k_{R}$, $k_{2}$ and $k_{3}$) decreased with the introduction of pre-treatment. As previously explained, the pre-treatment enhanced the solubilization of suspended solids. With more organic matter available, more time is needed to achieve the same percentage of degradation, leading to slower global process kinetics (Koch and Drewes, 2014).

**Table 3:** Optimal solution obtained for the parameters of the developed model and coefficient of determination for each test. Simulation of the *S*_*D*_ function with the experimental data from Donoso-Bravo et al. (2010).

	Experimental tests	
	Without pre-treatment	With pre-treatment
*S*_*max*_ (*mL*/*g*_*VS*_)	287.155	571.579
*k*_*L*_ (*d*^−1^)	0.076	0.047
*k*_*R*_ (*d*^−1^)	1.378	0.553
*k*_2_ (*d*^−1^)	0.892	0.094
*k*_3_ (*d*^−1^)	1.262	0.450
*α*	0.511	0.718
*R*^2^	0.999	0.999

#### 3.3.2 Performance comparison

After being applied in the interpretation of experimental data, the developed model was then compared with the most complete 1^st^ order model available in the literature, the 3-phase model (Deveci and Çiftçi, 2001), and the widely used Modified Gompertz model (Zwietering et al., 1990).

Structurally, the 3-phase model of Deveci and Çiftçi has the advantage of being composed by only 4 parameters, in comparison with the 6 parameters of the proposed model. Regarding the Modified Gompertz model, it offers the advantage of requiring only 3 parameters to estimate the cumulative production of biogas. However, it is not possible to obtain any information about substrate degradation or VFA accumulation profiles.

**Figure 8:**
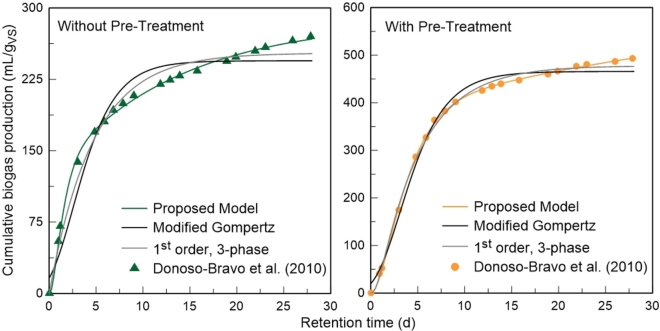
Simulation results obtained with the experimental data published by Donoso-Bravo et al. (2010), using the cumulative biogas production function of the 1^st^ order, 3-phase model (Eq. (3)), the Modified Gompertz model (Eq. (7)), and the proposed model (Eq. (15)).

In order to compare the performance of both models, equation (3) of the 1^st^ order, 3-phase model and equation (7) of the Modified Gompertz model were simulated with the same experimental data obtained from Donoso-Bravo study, as was done in the previous section for the proposed model. The results of these simulations are shown in Fig. 8, where it can be observed that the developed model allows a more accurate reproduction of the experimental information. It is therefore concluded that the introduction of the concept that the organic fraction of the substrate has different degradation speeds, has a significant impact on the results.

From Fig. 8, it is also possible to observe that in the beginning of the process, biogas production is not zero for the Modified Gompertz model, which is not realistic. This model was not designed from the process of anaerobic digestion, it is a re-parametrization of the original Gompertz model designed in the field of human mortality (Tjørve and Tjørve, 2017).

From Table 4 it can also be noticed that for both the 1^st^ order, 3-phase model and the Modified Gompertz model, the estimated values for the maximum potential of biogas production are inferior to the last value registered experimentally, which is obviously impossible. In addition to this, the optimal solution found for the Deveci and Çiftçi model parameters suggests very high values of $k_{3b}$ and $k_{3c}$, for the test without pre-treatment. According to these results, the optimal solution occurs when the concentrations of hydrolysis products and VFA compounds are practically zero throughout the process, which is not in agreement with reality.

**Table 4:** Optimal solution obtained for the parameters of the 1^st^ order, 3-phase model (Eq. (3)) and the Modified Gompertz model (Eq. (7)), and respective coefficient of determination for each test. Simulations performed with experimental data from Donoso-Bravo et al. (2010).

	Experimental tests	
	Without pre-treatment	With pre-treatment
1^st^ order, 3-phase		
$S_{max}$$\left(mL/g_{VS}\right)$	252.568	477.679
$k_{3a}$ ($d^{-1}$)	0.215	0.220
$k_{3b}$ ($d^{-1}$)	≫1	1.730
$k_{3c}$ ($d^{-1}$)	≫1	6.981
$R^{2}$	0.984	0.997
Modified Gompertz		
*P* ($mL/g_{VS}$)	244.463	465.702
*μ* ($mL/g_{VS}\cdot d$)	35.399	62.981
*λ* (*d*)	0	0.412
$R^{2}$	0.954	0.992

On the whole, it is possible to conclude that the developed model allows a more realistic and approximate reproduction of the kinetics of biogas production, during the process of anaerobic digestion.

#### 3.3.3 Predictive capacity

Empirical models require experimental data on biogas production to be able to estimate the biogas potential for infinite retention times. When properly calibrated, they can allow a reduction in the number of tests, as well as their duration (Weinrich et al., 2018).

**Figure 9:**
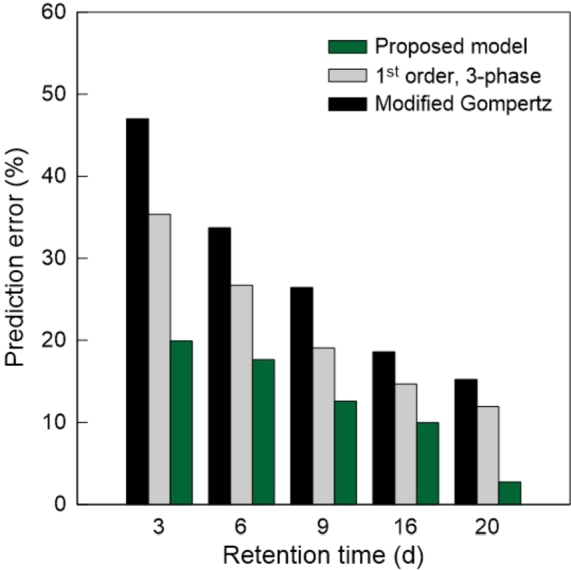
Prediction error at a given time *t* during the experiment expressed by $|S_{D}^{u}-y_{exp}^{u}|/y_{exp}^{u}$. Simulations performed with experimental data from Donoso-Bravo et al. (2010).

In order to evaluate the predictive capacity of the developed model in comparison with the 1^st^ order, 3-phase model and the Modified Gompertz model, simulations were performed for different instants during the experimental tests. Fig. 9 shows the results obtained for the test without pre-treatment, where the prediction error is the relative and absolute difference between the total measured biogas production ($y_{exp}^{u}$) and the respective estimate provided by the model ($S_{D}^{u}$) based on the available data at a given time *t*. For example, the prediction error on day 3 corresponds to the estimate made by the model based only on the experimental data of the first 3 days.

Throughout the test, the developed model provides systematically more accurate estimates compared to the other models. For a retention time of 20 days, with about 30% left to the end of the experiment, the model shows a prediction error of only 2.73%, suggesting that it is possible to obtain quite reasonable estimates within a shorter period of time. In fact, this pattern was verified for all the tests mentioned in this study. In Table 5 it is shown the time reduction that can be obtained using the different models, assuming as acceptable a prediction error under 5%.

**Table 5:** Time reduction obtained assuming a prediction error under 5%. Simulations performed for all case studies.

	Time reduction (%)		
	Proposed model	1^st^ order 3-phase	Modified Gompertz
Case study 1	58.3	33.3	0
Case study 2			
TS_1_	46.1	30.8	23.1
TS_2_	53.9	30.8	23.1
TS_3_	53.9	38.5	7.9
Case study 3			
Without pre-treatment	28.5	0	0
With pre-treatment	57.5	17.5	0

From Table 5 it can be found that the proposed model requires less experimental data to accurately estimate the total biogas production, leading to significant reductions in experimental time. For all the case studies, the model presented the best performance revealing a great flexibility and adaptability to different kinetic behaviours.

On the other hand, the Modified Gompertz model presents a lower predictive capacity in comparison with the 1^st^ order models. In many tests it was not even possible to reach a prediction error of less than 5% using all the experimental data. This model is more rigid because it is not based on the kinetics of the digestion process.

Considering the results obtained, the developed model presents itself as an interesting tool to reduce the number of laboratory tests as well as their duration.

## 4 Conclusions

The developed model allows to describe the process of anaerobic digestion in a more complete and precise way compared to other published empirical models. The assumption that the organic fraction of the substrate has different degradation speeds proved to be a good hypothesis. Given its accuracy in describing the biogas production kinetics, the model can be used as an auxiliary tool in determining the biogas potential, reducing the duration of laboratory tests through extrapolations for infinite retention times. Moreover, the model also provides information about the accumulation of VFA inside the reactor.

## Declarations

### Author contribution statement

Bruno Gouveia: Conceived and designed the experiments; Performed the experiments; Analyzed and interpreted the data; Wrote the paper. Elizabeth Duarte: Contributed reagents, materials, analysis tools or data. Aires dos Santos, Edgar Fernandes: Conceived and designed the experiments.

### Funding statement

This research did not receive any specific grant from funding agencies in the public, commercial, or not-for-profit sectors.

### Data availability statement

Data included in article/supplementary material/referenced in article.

### Declaration of interests statement

The authors declare no conflict of interest.

### Additional information

No additional information is available for this paper.
